# A guide to using functional magnetic resonance imaging to study Alzheimer's disease in animal models

**DOI:** 10.1242/dmm.031724

**Published:** 2018-05-18

**Authors:** Mazen Asaad, Jin Hyung Lee

**Affiliations:** 1 Department of Neurology and Neurological Sciences, Stanford University, Stanford, CA 94305, USA; 2 Department of Molecular and Cellular Physiology, Stanford University, Stanford, CA 94305, USA; 3 Department of Bioengineering, Stanford University, Stanford, CA 94305, USA; 4 Department of Neurosurgery, Stanford University, Stanford, CA 94305, USA; 5 Department of Electrical Engineering, Stanford University, Stanford, CA 94305, USA

**Keywords:** Alzheimer's disease, fMRI, Mouse models, Anesthesia, Resting state

## Abstract

Alzheimer's disease is a leading healthcare challenge facing our society today. Functional magnetic resonance imaging (fMRI) of the brain has played an important role in our efforts to understand how Alzheimer's disease alters brain function. Using fMRI in animal models of Alzheimer's disease has the potential to provide us with a more comprehensive understanding of the observations made in human clinical fMRI studies. However, using fMRI in animal models of Alzheimer's disease presents some unique challenges. Here, we highlight some of these challenges and discuss potential solutions for researchers interested in performing fMRI in animal models. First, we briefly summarize our current understanding of Alzheimer's disease from a mechanistic standpoint. We then overview the wide array of animal models available for studying this disease and how to choose the most appropriate model to study, depending on which aspects of the condition researchers seek to investigate. Finally, we discuss the contributions of fMRI to our understanding of Alzheimer's disease and the issues to consider when designing fMRI studies for animal models, such as differences in brain activity based on anesthetic choice and ways to interrogate more specific questions in rodents beyond those that can be addressed in humans. The goal of this article is to provide information on the utility of fMRI, and approaches to consider when using fMRI, for studies of Alzheimer's disease in animal models.

## Abstract (precis)

**Summary:** This Review examines the use of functional magnetic resonance imaging for studying Alzheimer's disease in animal models, and addresses important considerations concerning model choice, anesthetic use and application of stimuli.

## Introduction

Despite being discovered over 100 years ago and currently affecting millions of people, Alzheimer's disease (AD; see Glossary, Box 1) is a neurodegenerative disorder for which we still lack a clear mechanistic understanding and effective treatment options. In the United States alone, an estimated 5.4 million people have the disease, with this number expected to more than double by 2050 (Hebert et al., 2013). AD is the most common cause of dementia, with one new person in the United States receiving a diagnosis of the disease every 66 s on average (Alzheimer's Association, 2016).

**Box 1. Glossary**

**Alzheimer's disease (AD):** a neurodegenerative disease and the leading cause of dementia.

**Amyloid beta (Aβ):** a potentially toxic peptide characteristic of AD pathology.

**Amyloid precursor protein (APP):** the protein that can be cleaved to form Aβ.

**Antagonist:** a molecule that interferes with or inhibits another molecule or receptor.

**Autosomal dominant:** a pattern of genetic inheritance whereby an offspring can receive the gene from either parent with equal probability (‘autosomal’, in contrast to sex-linked genes) and having at least one copy of the gene is sufficient for phenotype manifestation in the offspring (‘dominant’, in contrast to recessive genes, which require two copies for phenotypic expression).

**Chemogenetic stimulation:** using chemogenetics as a means of activating a population of neurons.

**Chemogenetics:** with this technique, excitatory or inhibitory receptors that respond to a specific ligand are expressed in neurons of interest. The ligand is not normally expressed in the animal, so the neurons will only respond after it is injected.

**Default mode network:** a group of brain regions, the activities of which are highly correlated. The default mode network itself is active while the subject is awake but not attending to any specific task.

**Electroencephalography (EEG):** with this technique, electrodes placed on the scalp can record changes in electrical activity in the brain with high temporal resolution.

**Familial Alzheimer's disease (FAD):** a form of Alzheimer's disease caused by mutations in a specific set of genes (*APP*, *PSEN1* and *PSEN2*) that are passed down from parents to offspring in an autosomal-dominant manner.

**Fibers of passage:** neuronal fibers (axons or dendrites) originating from neurons, the cell bodies of which are not in the region of interest. Stimulation of these fibers can lead to activation of neurons outside of the region of interest and therefore confound analytical results by reducing the precision of the stimulation.

**Functional magnetic resonance imaging (fMRI):** with this technique, changes in brain activity over time are measured by using powerful magnetic fields to detect variations in cerebral blood flow, cerebral blood volume and/or cerebral blood oxygenation.

**GABA_A_:** a type of receptor that responds to gamma-aminobutyric acid (GABA), the main inhibitory neurotransmitter in the brain.

**Hemodynamic:** relating to changes in the blood as it moves through an organ, such as the brain.

**Neurovascular coupling:** the relationship between changes in neural activity and changes in the activity of nearby blood vessels.

**Optogenetic functional magnetic resonance imaging (ofMRI):** with this technique, researchers use fMRI to measure brain-wide changes in activity in response to optogenetic stimulation.

**Optogenetic stimulation:** using optogenetics as a means of activating a population of neurons.

**Optogenetics:** with this technique, light-activated ion channels can be used to activate or deactivate neurons with high temporal, spatial and cell-type specificity.

**Pittsburgh compound B (PiB):** a radioactive molecule that binds to Aβ plaques. It is commonly used in PET as a way to assess the amount of plaque in the brain of AD patients.

**Positron emission tomography (PET):** with this technique, a radioactive molecule is injected into the subject intravenously and the level of radioactivity in different parts of the body is measured noninvasively. A common application in AD involves the molecule PiB, which binds to Aβ plaques in the brain. By injecting PiB, one can then monitor Aβ plaque levels in the brain by measuring the amount of radioactivity that persists after a period of time.

**Signal-to-noise ratio (SNR):** a metric that compares the level of a desired signal in a measurement to the level of background noise.

**Sporadic Alzheimer's disease (SAD):** a form of Alzheimer's disease that patients develop without a previous family history of the disease.

AD and other dementias create a considerable physical, mental and emotional burden for both patients and caregivers, and this is exacerbated by the associated financial drain. In 2016 alone, the estimated cost of healthcare, long-term and hospice care for Americans over 65 with AD or other dementias was ∼$236 billion, with 68% coming from the US government and the rest from individuals. Furthermore, this number is expected to quadruple by 2050 (Alzheimer's Association, 2016). Given these facts, it is more necessary than ever for the clinical and research community to learn to understand, treat and prevent AD.

One tool that has proven invaluable for investigating AD is functional magnetic resonance imaging (fMRI; Box 1). In this approach, powerful magnetic fields are used to record changes in cerebral blood oxygenation, blood volume and blood flow as a way to measure brain activity while a patient performs tasks or is at rest (Logothetis et al., 2001; Ogawa et al., 1992). Because this technique is noninvasive and utilizes relatively common hospital equipment, researchers are using it to understand how the brains of AD patients change during disease progression (as discussed further below). The overarching goal is to use fMRI to gain a better understanding of how brain function is altered in AD, as well as to identify the changes that occur before symptom onset, with the aim of using such changes as predictive biomarkers. Such approaches are under investigation in both humans and animal models, with each offering distinct advantages.

The aim of this Review is to highlight the role of fMRI for studying AD in animal models. First, we discuss our current mechanistic understanding of this disease and the various hypotheses for its cause and propagation. Next, we review the numerous animal models of AD that are available and the differences between them. Finally, we examine the contributions of existing human and rodent fMRI studies to our current understanding of AD and the considerations involved in carrying out fMRI studies in animal models.

## Potential causes of AD

Many underlying mechanisms of AD have been proposed and tested, including amyloid plaques, neurofibrillary tangles, vascular abnormalities, dysregulation of cerebral glucose uptake and metabolism, and increased neuroinflammation (Table 1). Here, we briefly highlight the evidence for and against these contributors.

**Table 1.**
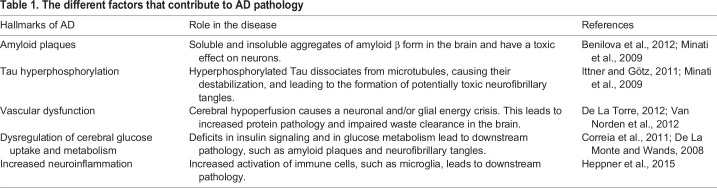
**The different factors that contribute to AD pathology**

The most common explanation for the etiology of AD is known as the amyloid cascade hypothesis. This hypothesis proposes that the accumulation of high levels of the amyloid β peptide (Aβ; Box 1) is the primary cause of the other pathologies observed in AD (Hardy and Higgins, 1992). Amyloid precursor protein (APP; Box 1) is a large membrane protein found in many tissues, including the brain, and it can be cleaved into different peptides (Zhang et al., 2012a). One of these, Aβ, can form aggregates and cause neuronal dysfunction if present in too high a concentration (Selkoe, 1991). According to the amyloid cascade hypothesis, the increased formation of Aβ in AD patients leads to toxic aggregates in the brain that cause and/or propagate disease pathology (Hardy and Higgins, 1992). Elevated Aβ levels in AD patients (Chen et al., 2014; Klunk et al., 2009; Villemagne et al., 2011, 2013), genetic determinants of AD that affect APP processing (Hardy and Higgins, 1992), and the neurotoxicity of Aβ plaques (Selkoe, 1991) are factors that all support the amyloid cascade hypothesis. However, clinical trials that have aimed to reduce Aβ levels in the brain have produced mixed results (Doody et al., 2014; Holmes et al., 2008; Salloway et al., 2014; Vellas et al., 2013). Moreover, individuals with normal cognition can have elevated Aβ levels (Chen et al., 2014; Klunk et al., 2009; Villemagne et al., 2011, 2013). It is therefore likely that while Aβ does play a key role in AD, it alone does not cause the disease.

Another potential contributor to the pathology of AD is the protein Tau (MAPT), which normally binds to microtubules. However, when Tau becomes hyperphosphorylated, it can break away from microtubules and form aggregates known as neurofibrillary tangles. Formation of these tangles and destabilization of microtubules owing to reduced Tau binding might cause neuronal dysfunction in AD. However, as with Aβ, hyperphosphorylated Tau does not seem to be the single causative factor in AD pathology (Minati et al., 2009). Notably, the most reliable biomarkers used to clinically track the progression of AD are levels of Aβ, phosphorylated Tau and total Tau in the cerebrospinal fluid of patients (Blennow et al., 2010). This suggests that a complex interplay of factors contributes to the disease state.

Adding to this complex picture are hypotheses that take a broader view of the disease. Some researchers consider AD to be similar to vascular dementia, where inadequate cerebral blood perfusion causes the associated symptoms (De La Torre, 2012; Van Norden et al., 2012). Others consider AD to be a form of diabetes caused by impaired insulin sensitivity and cerebral glucose utilization (Correia et al., 2011; De La Monte and Wands, 2008). Another hypothesis considers the contribution of increased inflammation in driving AD progression (Heppner et al., 2015). The hypotheses presented here are not mutually exclusive, and it is possible that AD is caused by many or all of these factors. A key consequence of our lack of understanding of the etiology of AD is our inability to produce an animal model that fully recapitulates human AD pathology, with which to study this devastating disease.

## Animal models of AD

In order to fully investigate the pathology that underlies a human disease, animal models that recreate one or more key aspects of that disease's pathology must first be established. In the case of AD, dozens of animal models have been generated and investigated over the years. Many of these replicate only a single aspect of the disease, such as amyloid plaque buildup or Tau hyperphosphorylation. Researchers can use these models to investigate the pathological impact from individual components of AD. Much of this modeling work has been performed in mice, although a few rat and nonrodent models of AD exist as well and will be briefly discussed. It is beyond the scope of this article to compare the available models in depth; we instead refer readers to several excellent, recent reviews on the topic (Chin, 2011; Do Carmo and Cuello, 2013; Hall and Roberson, 2012; Kaushal et al., 2013; Lecanu and Papadopoulos, 2013; Nazem et al., 2015; Salkovic-Petrisic et al., 2013). Here, we highlight the different types of animal models that are available for studying AD and the important issues to consider when selecting which model to work with.

### Models of AD

Alzheimer's disease can be divided into two major categories: familial (FAD) and sporadic (SAD; Box 1) Alzheimer's disease. Most cases of FAD are caused by numerous different mutations in the genes encoding amyloid precursor protein (*APP*) and presenilin 1 (*PSEN1*) and 2 (*PSEN2*), which form the γ-secretase complex that cleaves APP. These mutations are all inherited in an autosomal-dominant manner (Box 1). By contrast, SAD does not have a clear cause, although some predisposing factors have been identified, such as cerebrovascular dysfunction (De Toledo Ferraz Alves et al., 2010) and impaired insulin signaling in the brain (Correia et al., 2011). As a result, FAD is easier to study and to model than SAD, but unfortunately FAD only accounts for ∼5% of all AD cases and the mechanistic overlap between the two disease categories remains elusive (Minati et al., 2009).

Given that FAD is caused by mutations in *APP*, *PSEN1* or *PSEN2*, many transgenic mouse models of AD have been generated by inserting mutant human versions of these genes into the mouse genome. Similar models, using insertions of a mutant human *MAPT* gene, which encodes the Tau protein, also exist, leading to the formation of the neurofibrillary tangles that feature in AD. The promoters used for these transgenes are usually neuron specific, such as those from platelet-derived growth factor, or preferentially expressed in neurons, such as the hamster prion protein promoter (Chin, 2011; Hall and Roberson, 2012).

Mouse models of SAD also exist but are often considered less reliable for studying AD because of the poorly understood etiology of SAD. Given that SAD accounts for 95% of cases, many groups are working towards establishing better models of this more common form of AD. A promising example involves injecting streptozotocin, a compound used to induce diabetes in animal models, into the lateral ventricles of rodent brains. This causes insulin resistance in the brain, which might play a key role in SAD pathology (Correia et al., 2011; Gao et al., 2013; Grieb, 2015; Salkovic-Petrisic et al., 2013). Mice generated by this method have been compared to the 3xTg-AD line (Table 2), which is a transgenic mouse model that contains mutations in *APP*, *PSEN1* and *MAPT*. Both models show altered expression levels of synaptic proteins, increased levels of hyperphosphorylated Tau in the brain, changes in insulin signaling, and neuroinflammation (Chen et al., 2012a). Critically, both models also share learning and memory deficits (Chen et al., 2012a) and have similar gene expression profiles in the hippocampus and cortex (Chen et al., 2012b). While these models are not identical, the similarities between them indicate that it is possible to recapitulate certain aspects of SAD in mouse models by using chemical compounds (Kaushal et al., 2013).

**Table 2.**
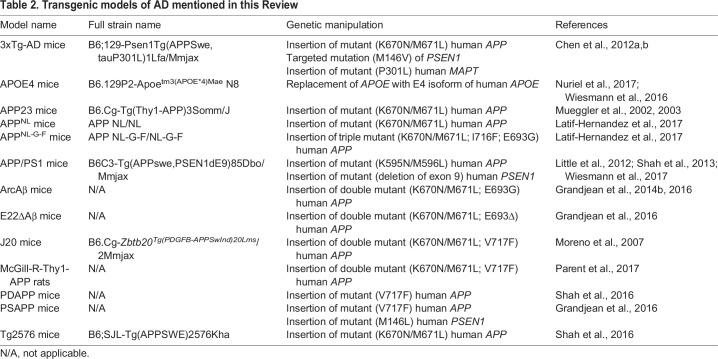
**Transgenic models of AD mentioned in this Review**

There are also a number of nonmurine models of AD that can be studied with fMRI. Transgenic rats for AD exist, although their pathology is subtle compared with that of transgenic mice, with similar models in mice producing more amyloid plaques at an earlier age (Do Carmo and Cuello, 2013). Researchers interested in studying the progression of the disease prior to cognitive symptom onset could find it advantageous to use models that progress more slowly, whereas those testing methods of clearing plaques might opt for faster-progressing models. Induced rat models include the ferrous amyloid buthionine model, which is generated by co-injecting Aβ into the brain with ferrous sulfate and buthionine sulfoximine, compounds that increase the toxicity of Aβ (Lecanu and Papadopoulos, 2013). Other animal models can be developed by triggering neuroinflammation, using agents such as lipopolysaccharide (Nazem et al., 2015). Additionally, features of spontaneous AD, such as Aβ accumulation and neurofibrillary tangles, have been found in the brains of aging individuals of some species, including dogs, degus, sheep, rabbits, bears and nonhuman primates (Kaushal et al., 2013).

### Inconsistencies between AD models

One key concern about animal models of AD is the variability in the disease phenotypes they exhibit. Key features of the disease, such as cognitive deficits, amyloid plaques, neurofibrillary tangles, neurodegeneration and synaptic dysfunction, can vary greatly between models, as well as the age of disease onset (Chin, 2011; Do Carmo and Cuello, 2013; Hall and Roberson, 2012; Nazem et al., 2015; Puzzo et al., 2014). Additionally, a comparison of males and females from within different strains of AD model mice showed significant differences in longevity (Rae and Brown, 2015). When studying AD, researchers should therefore not only consider which AD model to use, but also whether males and females have different progression timelines for the symptoms of interest.

Many groups have tried to reconcile these differences between models in an effort to understand whether common AD symptoms cause AD. For example, a meta-analysis of research results derived from different strains of transgenic mice found that only a weak association exists between high Aβ levels and decreased cognitive function (Foley et al., 2015). Moreover, fMRI studies conducted on AD mice in the resting state indicate that detected changes in functional connectivity depend more on the mouse model used than the levels of Aβ in the brains of the animals (Grandjean et al., 2016). A meta-analysis of human studies looked at how decreased cognitive performance is related to high Aβ levels as measured by different methods (Hedden et al., 2013). The authors' primary analysis focused only on studies that measured Aβ levels via the common method of Pittsburgh compound B (PiB) positron emission tomography (PET; Box 1). These analyses found that increased Aβ levels are associated with decreased performance in episodic memory, but not in executive function, global cognition, working memory, processing speed, visuospatial function or semantic memory. However, executive function and global cognition did show significant deficits when including studies that measured Aβ levels using different methods, such as blood plasma (Hedden et al., 2013). One potential explanation for these findings is that Aβ is indeed toxic, but not because it forms insoluble plaques. Instead its toxicity might be caused by soluble oligomers that also form from the aggregation of Aβ, although the exact relationship between soluble and insoluble Aβ aggregates is unclear (Benilova et al., 2012; Mroczko et al., 2017). These oligomers can form before the Aβ plaques do, and their presence correlates with increased neuroinflammation, oxidative stress, neuronal dysfunction and cognitive deficits (Zhang et al., 2012b,c), pointing towards a new candidate in the AD puzzle and further complicating the researchers' choice of animal model.

### Choosing a model

The fact that animal models of AD are so different can make it difficult to discern the underlying mechanism(s) of the disease. It also calls into question how faithfully these models can recapitulate human pathology, which itself can be variable. This disconnect could explain why some treatments that improve cognition in animals fail to do so in human patients (Doody et al., 2014; Holmes et al., 2008; Salloway et al., 2014; Vellas et al., 2013). It also means that researchers who are studying AD need to carefully choose the model that best suits their study aims. In choosing a model, one should consider several important questions (Box 2). One approach is to investigate several models in parallel to test how well a set of findings can be generalized.

**Box 2. Questions to consider when choosing an AD model**

Which aspects of AD are most relevant to the planned study?

Is a specific gene, protein or symptom of particular interest?

Do symptoms in certain models progress too quickly or too slowly for the experimental design?

Is the sex of the mouse or rat important?

Is the background species or strain important?

Are induced or spontaneous models of AD preferable to transgenics?

Despite these concerns, there is still tremendous value in studying animal models of AD. Although some might only recapitulate certain aspects of the human disease, such models make it possible to tease apart the specific contribution that a single pathological change can contribute to disease progression. Experimental techniques, such as optogenetics (Box 1) (Boyden et al., 2005; Lee et al., 2010) and electrophysiology, can be used to induce and measure pathological changes that cannot be investigated in humans. Animal models can also be used to test novel drugs and therapies. Finally, when working with animal models, researchers know which animals will develop AD before symptoms appear, supporting the discovery of new disease biomarkers or prophylactic treatments. When used properly, animal models are a vitally important tool for understanding the underlying pathology of AD and can be effectively used in fMRI-based studies, as discussed later in this Review.

## Clinical fMRI studies of AD

Human fMRI studies of AD have led to some promising findings in recent years. These studies can be broadly divided into two categories: those examining brain network activity during the resting state (so-called resting state fMRI) (Chhatwal and Sperling, 2013; Dennis and Thompson, 2014; Dickerson and Sperling, 2008; Liu et al., 2008; Weiner et al., 2012) and those that measure responses to specific tasks or stimuli (Chhatwal and Sperling, 2013; Dickerson and Sperling, 2008; Li et al., 2015; Sugarman et al., 2012; Weiner et al., 2012).

Resting state fMRI in AD patients has revealed decreased functional connectivity between numerous cortical brain regions and the hippocampus (Greicius et al., 2004; Sheline et al., 2010). Decreased connectivity has also been seen in the default mode network (Box 1), specifically in brain regions such as the posterior cingulate and medial prefrontal cortices (Sorg et al., 2007; Zhang et al., 2009). Conversely, some brain regions exhibit increased connectivity, which some believe to be caused by compensatory mechanisms in the brains of AD patients (Bäckman et al., 2000; Grady et al., 2003; He et al., 2007; Prvulovic et al., 2002; Zhang et al., 2009). Activity changes in the default mode network have also been used to build predictive models of the progression from mild cognitive impairment to AD (Petrella et al., 2011). The authors compared the default mode networks from patients with mild cognitive impairment with those from healthy controls. They followed up with the the patients a few years later, and found that those patients whose default mode networks were least similar to those of healthy controls were more likely to have progressed to AD (Petrella et al., 2011).

In addition to resting state imaging, fMRI can be performed while patients undertake various tasks. For instance, during a visual discrimination task, AD patients were found to have increased activity in the occipitotemporal cortex and decreased activity in the superior parietal lobule relative to healthy controls (Prvulovic et al., 2002). In another study using a verbal encoding and recognition task, the AD group had reduced activation in the medial temporal lobe and increased activation in the left prefrontal brain regions relative to healthy controls (Rémy et al., 2005). But, even though proven fruitful for studying AD, fMRI also has important limitations that need to be considered.

## Confounding factors in fMRI studies

As a clinical imaging tool, fMRI is both powerful and widespread, and able to show changes in brain activity during specific tasks and/or while the patient is at rest. The key advantages of this imaging technique include its noninvasive nature and its ability to image the entire brain. These benefits do, however, come with some tradeoffs. For instance, fMRI is a relatively slow method, measuring responses in the order of seconds compared with the millisecond precision of neuronal firing (Lee et al., 2010).

Although fMRI is often used to indirectly measure neural activation, other factors can affect the observed signals and must be considered (Uludağ and Blinder, 2018). This is partly because many parameters, such as magnetic field strength, can vary between studies, and changes in these parameters can result in different temporal or spatial brain activation patterns. Additionally, because fMRI relies on hemodynamic measurements (Box 1) as a proxy for neural function (Logothetis et al., 2001; Ogawa et al., 1992), conditions that affect blood flow can lead to an incorrect interpretation of the observed changes in brain activity. Cerebral amyloid angiopathy is one such condition associated with AD, whereby Aβ buildup on cerebral blood vessels leads to impairments in vascular dilation, to a degree that is detectable by fMRI (Princz-Kranz et al., 2010). Other vascular pathologies in AD (Klohs et al., 2014) include decreased vascular density (Ielacqua et al., 2016), reduced cerebral blood volume (Zerbi et al., 2013) and cerebral hypoperfusion (Weidensteiner et al., 2009). When interpreting fMRI results, it is therefore important to consider how both neural and vascular factors are contributing to the changes observed. This concerns comparisons between different animal models, but also when comparing human and rodent fMRI studies.

Alternatively, electroencephalography (EEG; Box 1) can be used to measure brain activity at a higher temporal resolution than fMRI, but at a lower spatial resolution (Engel et al., 2005). Implantable electrodes can record signals from specific areas of the brain, thereby increasing spatial resolution, but are invasive (Engel et al., 2005). Tools such as PET (Box 1) can be used to measure metabolic function in the brain via the injection of radioactive compounds, such as 18-fluorodeoxyglucose, but this method also has low temporal resolution and is invasive, owing to the injection of a radioactive substance (Nordberg, 2004). That said, a variety of measuring methods can be used in parallel to paint a more complete picture of AD.

## Utilization of fMRI in animal models of AD

**Fig. 1.**
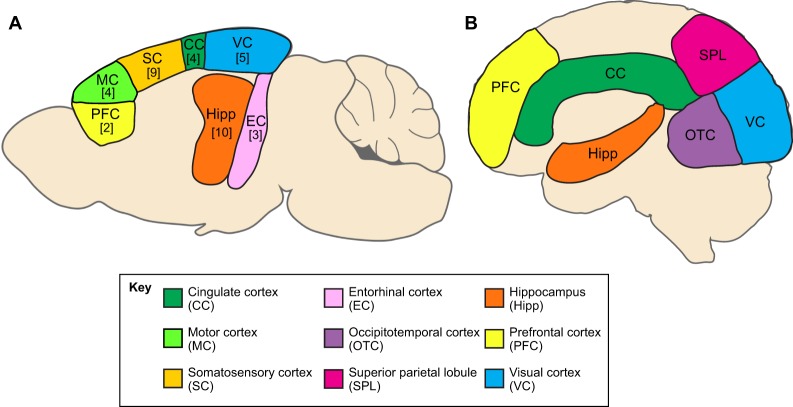
**Schematic of rodent and human brain regions commonly identified in fMRI studies of AD.** (A,B) Locations of brain regions in which fMRI detects changes in AD in rodent (A) and human (B) subjects. Regions are shown overlayed on a sagittal view of the brain. Numbers in brackets in A indicate the number of published studies that found a particular brain region to be affected in animal models of AD by using fMRI; specifically, the cingulate cortex (CC) [4] (Grandjean et al., 2016; Mueggler et al., 2002; Parent et al., 2017; Shah et al., 2016); the entorhinal cortex (EC) [3] (Little et al., 2012; Moreno et al., 2007; Nuriel et al., 2017); the hippocampus (Hipp) [10] (Grandjean et al., 2016; Latif-Hernandez et al., 2017; Little et al., 2012; Moreno et al., 2007; Nuriel et al., 2017; Parent et al., 2017; Shah et al., 2013, 2016; Wiesmann et al., 2016, 2017); the motor cortex (MC) [4] (Grandjean et al., 2014b; Mueggler et al., 2002; Wiesmann et al., 2016, 2017); the prefrontal cortex (PFC) [2] (Latif-Hernandez et al., 2017; Grandjean et al., 2016); the somatosensory cortex (SC) [9] (Grandjean et al., 2014b, 2016; Little et al., 2012; Mueggler et al., 2002, 2003; Sanganahalli et al., 2013; Shah et al., 2013; Wiesmann et al., 2016, 2017); and the visual cortex (VC) [5] (Grandjean et al., 2016; Little et al., 2012; Mueggler et al., 2002; Wiesmann et al., 2016, 2017).

Much of the fMRI research into AD has been performed on humans, but more studies in rodent models of AD have started to incorporate fMRI in recent years. A common deficit observed in human AD is decreased functional connectivity between the cortex and the hippocampus relative to healthy controls (Greicius et al., 2004; Sheline et al., 2010). As discussed below, this is also a common finding in rodent AD studies, suggesting that similar changes could be occurring in the brains of both species (Fig. 1). However, no fMRI-based AD studies have directly compared rodents and humans. In an ideal scenario, researchers would conduct the same experiment and analysis in AD patients and a comparable AD rodent model. This could be achieved by looking only at patients with specific mutations of the *APP* gene and at transgenic mice with the insertion of the same mutant allele. These types of studies would be valuable for analyzing, from the perspective of fMRI, how well animal models recapitulate the changes seen in the clinic.

Even without direct comparisons, AD rodents offer potential for insights beyond what is achievable in the clinic. Since researchers know which animals will develop AD before symptom onset, they can identify changes that precede common pathological and cognitive symptoms (Grandjean et al., 2014b; Latif-Hernandez et al., 2017; Shah et al., 2016). These changes could be used as biomarkers to screen patients for AD and provide early treatment options. Using animal models can also inform the mechanism of AD progression by using models that isolate specific aspects of AD, such as Aβ plaques, or techniques not available in humans to examine specific networks in the brain, such as optogenetics (discussed below). Being able to learn more about the underlying mechanisms of AD will be crucial for interpreting the results that we see in the clinic. The studies highlighted below have identified that deficits similar to those in patients are seen in animal models of AD, but more research is needed to leverage the unique advantages of animal models.

### Existing studies using fMRI in AD animal models

**Fig. 2.**
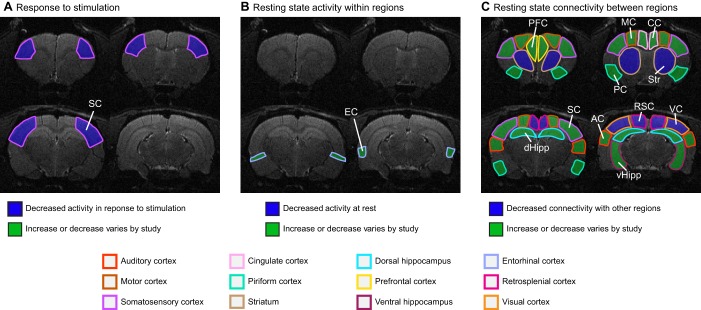
**Regions found to be affected by AD in rodent fMRI studies.** (A-C) The highlighted areas, overlayed onto MRI images of normal rodent brains, represent the brain regions that show different activities in fMRI studies of AD (summarized in Table 3). For clarity, the study results are divided into three groups: those that investigated the response to a specific stimulus (A), those that looked at changes within a single specific brain region during the resting state (B), and those that looked at changes in functional connectivity between brain regions during the resting state (C). AC, auditory cortex; CC, cingulate cortex; dHipp, dorsal hippocampus; EC, entorhinal cortex; MC, motor cortex; PC, piriform cortex; PFC, prefrontal cortex; RSC, retrosplenial cortex; SC, somatosensory cortex; Str, striatum; VC, visual cortex; vHipp, ventral hippocampus.

Studies using fMRI in animal models of AD have already shown some promising results (Table 3, Fig. 2). One study utilized fMRI in the ferrous amyloid buthionine rat model (discussed above). These rats had reduced activation in the somatosensory cortex in response to forepaw electrical stimulation relative to control rats (Sanganahalli et al., 2013). Another study used fMRI in the J20 mouse model of AD (Table 2) and reported that these mice have reduced cerebral blood volume in the entorhinal cortex at rest compared with wild-type littermates. Moreover, daily administration of the amyloid-lowering drug flurbiprofen gradually increased cerebral blood volume in the entorhinal cortex of these mutant mice over a period of 5 weeks (Moreno et al., 2007). Further, electrical stimulation on the hindpaw of APP23 mice (Table 2) resulted in reduced somatosensory cortex activity as measured by fMRI compared with that of control mice (Mueggler et al., 2003). Additionally, fMRI has shown alterations in the cerebral hemodynamic response. The authors found that cerebral blood volume in the somatosensory cortex normally shows large increases following intravenous infusion of the GABA­_A_ antagonist (Box 1) bicuculline, but this effect was reduced and delayed in the APP23 AD mice (Mueggler et al., 2002). This suggests that both neuronal and vascular activities might be significantly compromised in AD.

**Table 3.**
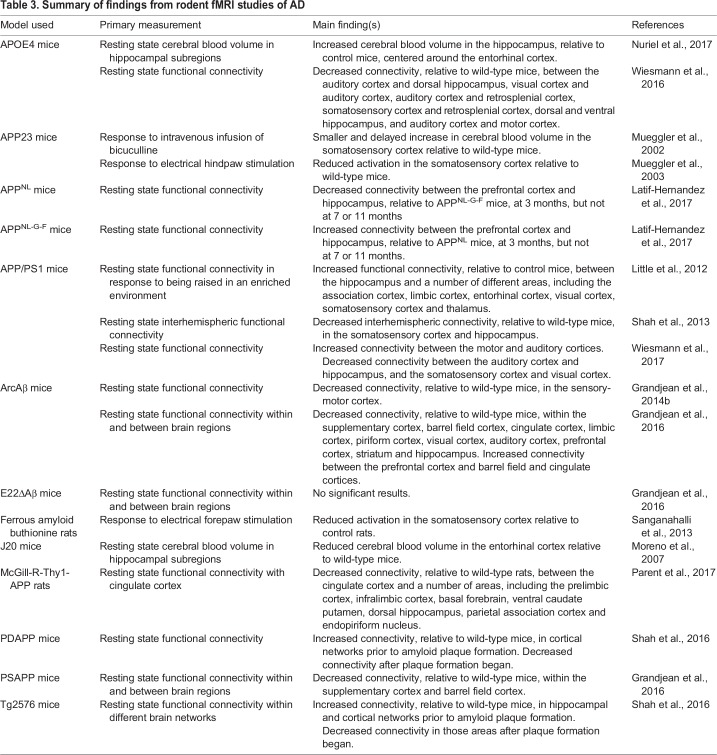
**Summary of findings from rodent fMRI studies of AD**

Unlike in humans, not many studies looking at functional connectivity in resting state networks exist in AD rodents, although more have been performed recently (Grandjean et al., 2014b, 2016; Latif-Hernandez et al., 2017; Little et al., 2012; Nuriel et al., 2017; Parent et al., 2017; Shah et al., 2013, 2016; Wiesmann et al., 2016, 2017). One such study in the McGill-R-Thy1-APP rat model (Table 2) found decreased functional connectivity between the cingulate cortex and other areas of the cortex and hippocampus relative to wild-type rats (Parent et al., 2017). Another group showed that APOE4 mice (Table 2) have decreased functional connectivity between different cortical regions as well as between the cortex and hippocampus (Wiesmann et al., 2016). When the animals were at rest, the APOE4 mice also had increased activity in the entorhinal cortex (Nuriel et al., 2017).

One study found that APP/PS1 mice (Table 2) have a decreased interhemispheric functional connectivity in the somatosensory cortex and hippocampus relative to their wild-type littermates (Shah et al., 2013). Another group looked at APP/PS1 mice raised in an enriched environment with toys and running wheels for 2 months (Little et al., 2012). They found increased functional connectivity between the hippocampus and cortex in the enriched group relative to APP/PS1 mice that grew up in a standard environment. It has also been shown that these mice have decreased functional connectivity between the auditory cortex and the hippocampus, as well as between the somatosensory and visual cortices, relative to wild-type animals (Wiesmann et al., 2017). Interestingly, the same analysis found increased functional connectivity between the motor and auditory cortices in these mice.

Shah et al. tracked functional connectivity in the brains of Tg2576 and PDAPP mice (Table 2) relative to their wild-type littermates as they aged (Shah et al., 2016). The authors found increased functional connectivity throughout the brain in both models prior to plaque development, and then a decrease in functional connectivity after plaques had begun forming when the mice were older. One group looking at the ArcAβ mouse model (Table 2) showed decreased functional connectivity in the sensory-motor cortex prior to plaque formation (Grandjean et al., 2014b). A later study compared functional connectivity within brain regions of E22ΔAβ, PSAPP and ArcAβ mice relative to wild-type mice (Grandjean et al., 2016). They found that the PSAPP model showed a decrease in functional connectivity in the supplementary and barrel field cortices. Additionally, the ArcAβ mice showed decreased functional connectivity within several different cortical subregions, and the E22ΔAβ mice showed no differences (Grandjean et al., 2016).

One interesting study looked at functional connectivity between the prefrontal cortex and the hippocampus in two slightly different models of AD, APP^NL-G-F^ and APP^NL^ mice (Table 2), the difference between them being that the latter has only one *APP* mutation, whereas the former has three (Latif-Hernandez et al., 2017). They compared prefrontal-hippocampal connectivity at 3, 7 and 11 months of age between the two and found that APP^NL-G-F^, the AD model that produces more Aβ plaques, has increased connectivity at 3 months, but that difference disappears by 7 and 11 months of age.

Taken together, these studies confirm that resting state fMRI in rodents is a meaningful approach for AD research, with the most commonly observed deficits being decreased functional connectivity in the hippocampus and/or cortex. That said, when performing fMRI in rodents, a number of considerations need to be accounted for beyond the typical challenges of its use in humans (as briefly discussed above and reviewed in Gozzi and Schwarz, 2016; Pan et al., 2015). fMRI has revealed that rats require appropriate levels of habituation and restraint in order for their default mode network to resemble that of humans when awake, which highlights potential confounding factors (Upadhyay et al., 2011). The lack of cooperation of the animals also requires the use of either anesthesia or restraint, which can be stressful and alter brain physiology if not used judiciously. Even when animals are kept still, a small amount of motion might occur, which can affect image quality. Additionally, the significantly smaller size of the rodent brain requires image acquisition at a higher spatial resolution, which comes at the cost of a lower signal-to-noise ratio (SNR; Box 1) (Baltes et al., 2009). Lastly, researchers might also want to examine specific networks, which is typically achieved in humans by having subjects perform behavioral tasks while in the scanner, but is much more difficult to implement in animal models. Some of these concerns, such as anesthesia, low SNR (Box 3) and examination of specific brain networks, can be systematically addressed to minimize their impact on study outcomes.

**Box 3. Improving signal with cryogenic radiofrequency coils**

Given the size of the rodent brain, obtaining high quality fMRI images with good spatial resolution can be challenging. Radiofrequency (RF) coils placed near the head of the animal detect the signal during image acquisition in an MRI scanner. Opting for cryogenically cooled RF coils can be one way to improve image quality by reducing the thermal noise in the coil. This is especially true for fMRI performed on small animals (compared with humans) because, while both coil and sample noise can affect the signal-to-noise ratio (SNR), coil noise becomes more dominant as coil size decreases (Junge, 2012). An *in vivo* mouse brain fMRI study that compared cryogenic RF coils with coils at room temperature showed that the SNR of the cryogenically cooled coils was up to 2.5 times higher (Baltes et al., 2009). This, in turn, can improve the spatial resolution and reduce image acquisition time, both of which are favorable parameters in fMRI.

Many groups have used cryogenic RF coils for fMRI in mice and found it advantageous for a variety of applications, including resting state measurements (Mechling et al., 2014), somatosensory stimulation (Baltes et al., 2011; Bosshard et al., 2010, 2012; Reimann et al., 2016; Schroeter et al., 2014) and optogenetic stimulation (Takata et al., 2015). Although undoubtedly advantageous, RF coils can be prohibitively expensive and as such are not always available to researchers. Additionally, cryogenically cooled RF coils are most effective when they can be placed as close as possible to the sample (Niendorf et al., 2015). This can be a limitation if the mouse's skull contains implantations for other experiments, such as electrophysiology or optogenetics. It also limits the possibility of stable head fixation for awake imaging, although using a custom cradle in a recently published study provided a potential solution (Yoshida et al., 2016).

### Anesthesia and its effects on fMRI

The use of anesthesia is a primary concern when imaging small animals. Although it is required to keep the animals still during imaging sessions, as motion can ruin the images, anesthesia can alter the physiology or brain activity of treated animals to the point where the results obtained are no longer representative of the awake state (Desai et al., 2011; Liang et al., 2012, 2015; Nasrallah et al., 2012, 2014c).

An alternative approach is to restrain the animal, but this induces stress, which itself can be a confounding factor. That said, resting state fMRI in awake rats has been successfully performed after habituation to a restraint apparatus (Upadhyay et al., 2011). However, the authors note the possibility that the animals learned helplessness rather than becoming habituated to the restraint. If the rats were indeed in a state of chronic stress owing to learned helplessness, this could mimic habituation, but affect their physiology and brain network activity. As such, Upadhyay and colleagues recommend further behavioral testing for learned helplessness, as well as measurements of blood corticosterone, a marker of stress (Upadhyay et al., 2011). Another group performed awake fMRI in mice during cued fear conditioning. This group tested two different habituation protocols of either 5 or 12 days (Harris et al., 2015). They measured blood corticosterone, body weight loss, body movements during scanning and respiration rate as indicators of stress. Using these metrics, they determined that the 12-day habituation protocol was considerably superior to the 5-day protocol in reducing stress to near-baseline levels during scanning. Based on these studies, it appears that awake imaging in rodents is possible if researchers carefully account for the potentially confounding effects of stress.

**Fig. 3.**
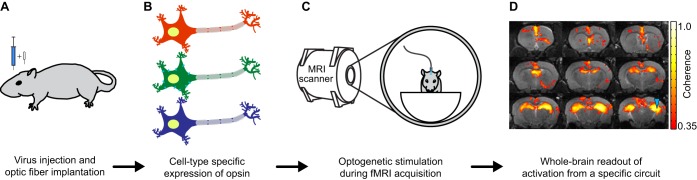
**Schematic of optogenetic fMRI experiments.** (A) First, rodents are injected with a viral vector that induces neurons to express opsins, light-responsive membrane-bound proteins that, when expressed, allow light to trigger or inhibit neural firing. The animals are also implanted with an optic fiber that allows light to be delivered to a specific region of the brain. (B) The opsin can be expressed in specific cell types, shown here as different colored neurons. Note that only the green neuron is expressing the opsin, as highlighted by the blue proteins in the membrane of the cell. Cell-type-specific targeting allows researchers to focus on specific cell types, such as cholinergic neurons, a population known to be degenerated in AD, in order to better understand the downstream effects of cholinergic dysfunction. (C) Opsin-expressing neurons are stimulated with light during fMRI in order to visualize the resulting circuit activity throughout the whole brain. (D) Example of ofMRI data from wild-type rats showing the response of hippocampal neurons to optogenetic stimulation. Higher coherence values indicate stronger activation in that region. The location of the optic fiber is indicated by the blue arrowhead.

If anesthesia is to be used, it is important to consider drug type and dosage, as both factors can influence brain activity. In rats receiving optogenetic stimulation (Box 1, Fig. 3) to the medial prefrontal cortex, the downstream brain activity, as measured by fMRI, was reduced in animals treated with 1-1.5% isoflurane, as compared with awake animals (Liang et al., 2015). Despite significant overlap in the brain regions that were activated in the awake and anesthetized states, there were some discrepancies as well. These included activation in the lateral hypothalamus, medial septum and mediodorsal nucleus of the thalamus in the awake rats, but not in the anesthetized rats (Liang et al., 2015). In addition to these findings, rats treated with 2% isoflurane showed weakened and altered connections in resting state fMRI compared with untreated rats imaged in the awake state (Liang et al., 2012). Following optogenetic activation, somatosensory cortex activity in mice treated with 0.7% isoflurane showed similar differences to those reported by Liang et al. (2012), when compared with the awake state (Desai et al., 2011). In another study that used medetomidine as a sedative for resting state fMRI in mice, researchers observed a dose-dependent decrease in functional connectivity in the brain (Nasrallah et al., 2014c). Furthermore, the impact of a given dose of medetomidine on functional connectivity varied between brain regions, an effect previously shown in rats (Nasrallah et al., 2012).

Thus, it is important to determine which anesthetic or habituation protocol is best suited for a particular study. Certain anesthetics can affect specific brain areas in different ways, so the decision of which anesthetic to use should be partially informed by the brain regions of interest, and by whether the response is to be recorded during activity, at rest or in response to a specific stimulus. For instance, one study measured interhemispheric functional connectivity in mice (Grandjean et al., 2014a). They found that, when using isoflurane, propofol or urethane as the anesthetic, connectivity was apparent in the cortex, but not in the striatum. By comparison, when using medetomidine, the authors found greater interhemispheric striatal connectivity, and reduced connectivity in the cortex (Grandjean et al., 2014a). Given the potential vascular abnormalities in AD animals relative to healthy controls, the effects that anesthetics have on neurovascular coupling (Box 1) might also be different in AD models. An anesthetic's mechanisms of action, and whether it acts as a vasodilator or vasoconstrictor, should also be considered, as vascular activity is central to fMRI measurements (Ogawa et al., 1992). Beyond anesthetic choice, other parameters can influence fMRI readouts, such as the partial pressure of carbon dioxide and oxygen in the blood, as shown for the somatosensory response and resting state brain networks in rats (Nasrallah et al., 2015). In all cases, the anesthetic dose used and the time taken to image the animals' brains should be minimized in order to mitigate some of these effects. We also suggest testing several anesthetics in parallel to assess the consistency of the results.

### Investigating specific brain circuits

In human studies of AD, both resting state and task-based fMRI have been informative (as discussed previously), whereas much of the rodent fMRI work for AD has focused on the resting state. This is largely because training animals to perform specific tasks is a difficult and time-consuming process, with the challenges only exacerbated by trying to implement the task in the fMRI scanner. The space constraints of the scanner, the stressful environment, and restrictions on magnetic materials also limit the options for performing task-based fMRI in animals. To our knowledge, no published studies exist in which AD rodents have performed tasks during fMRI. However, by not performing such task-based imaging, researchers potentially miss a wealth of information that could be translationally relevant. Although it is difficult to use task-based fMRI in animal models as a means to investigate specific brain circuits, an alternative set of tools are available, as we discuss below.

In an ideal scenario, it would be possible to have mice perform behavioral tasks similar to those that AD patients perform, while awake, being imaged, with their heads fixed, and calm. A number of groups have achieved this goal with optical imaging by using virtual reality environments. Rats and mice have been trained to navigate virtual environments, even with their heads restrained, although the setups require specialized equipment, such as spherical treadmills and projector screens (Aronov and Tank, 2014; Dombeck et al., 2010; Hölscher et al., 2005). The visual environment can also be replaced by a tactile one. In a recent study, researchers placed two movable walls on either side of a mouse's whiskers (Sofroniew et al., 2014). They were then able to guide mice walking on a spherical treadmill based on how much pressure each wall exerted on the whiskers, indicating to the mouse how sharply to turn in the virtual corridor. While these experiments show promise, coupling them with the fMRI scanner would require the development of new hardware that is both small and completely fMRI compatible. Visuospatial deficits are common in AD (Binetti et al., 1998; Kaskie and Storandt, 1995), and combining spatial tasks with fMRI would be useful for better understanding how changes in brain activity relate to these deficits as the disease progresses.

A different way that researchers can examine different brain circuits in rodents is by using external stimuli. Heat (Reimann et al., 2016) or electrical stimulation (Adamczak et al., 2010; Ahrens and Dubowitz, 2001; Baltes et al., 2011; Bosshard et al., 2010; Goloshevsky et al., 2011; Huttunen et al., 2008; Mueggler et al., 2003; Nasrallah et al., 2012, 2014a,b, 2015, 2017; Pelled et al., 2007) are among the most commonly used stimuli. The stimulus is applied to a paw and the subsequent changes in brain activity are measured. Such simple somatosensory and pain tests can easily be replicated in humans for comparison. Memory deficits, which are central to AD, can also be tested in this way. By pairing electric shocks with visual stimulation in the scanner, a fear-conditioning paradigm has been established for awake fMRI in mice (Harris et al., 2015). This combined approach – fear conditioning with fMRI – could be used in AD research to identify brain circuit changes during memory tasks. Researchers could choose other forms of stimuli to measure sensory responses or memory, although their use should be carefully planned. For instance, the noisy scanner environment can potentially drown out auditory stimuli.

Another approach would be to combine local electrical stimulation with fMRI. By implanting magnetic resonance imaging (MRI)-compatible electrodes into specific parts of the brain, researchers can visualize the brain-wide response to local stimulation of specific neurons. This technique has been used to examine the effect of different stimulation paradigms on hippocampal (Angenstein et al., 2007; Canals et al., 2008; Helbing et al., 2013) and thalamic (Yang et al., 2013) network activity in rats. It has also been used to measure responses to deep brain stimulation in rats (Lai et al., 2013; Van Den Berge et al., 2015, 2017), a technique that is currently being used in some AD patients to mitigate cognitive decline (Laxton and Lozano, 2013). Electrical stimulation in combination with fMRI is a powerful approach, as it permits the targeting and activation of brain circuits, with a wide range of stimulation frequencies and patterns chosen by the researcher to best suit a specific experiment or therapy. However, without a way to target specific cell types, all local neurons can be stimulated indiscriminately, including fibers of passage (Box 1). If specific subpopulations of neurons are to be investigated, a stimulation method that utilizes targeted genetic expression, such as opto- or chemogenetic stimulation (Box 1), would be more appropriate. For example, it is known that cholinergic dysfunction is a common feature of AD (Mesulam, 2004). Being able to specifically target cholinergic cells (Fig. 3) would allow researchers to investigate the role of the neurotransmitter acetylcholine in the progression of AD.

### Cell-type-specific neuromodulation

Optogenetic activation of neurons can be combined with fMRI in so-called optogenetic fMRI (ofMRI; Box 1, Fig. 3) (Lee et al., 2010). In this approach, researchers can activate specific neural circuits and examine the whole brain's response in real time (Fang and Lee, 2013). This recently developed method is gaining traction owing to the amount of control and precision it offers, having been applied to both rats (Duffy et al., 2015; Liu et al., 2015; Weitz et al., 2015) and mice (Desai et al., 2011; Lee et al., 2016). Compared with external stimuli, optogenetic stimulation has the advantage of targeting specific brain circuits based on their anatomical location (Weitz et al., 2015), cell type (Lee et al., 2016) and the stimulation frequency used (Liu et al., 2015). Optogenetic stimulation of the hippocampus has even been used to reduce amyloid plaque levels in AD mice *in vivo* (Iaccarino et al., 2016).

Although not yet applied to AD models, ofMRI could be a powerful tool for the future of AD research. For instance, fMRI of AD patients has shown hippocampal dysfunction relative to healthy controls (Liu et al., 2008), and a number of ofMRI studies have already been conducted that stimulate this brain region in rodents (Duffy et al., 2015; Takata et al., 2015; Weitz et al., 2015). Performing ofMRI in the hippocampus of AD rodents could help us understand how AD affects other parts of the brain that receive signals from the hippocampus. ofMRI has also been used to investigate brain-wide activation resulting from dopaminergic neuron stimulation (Lee et al., 2016). A similar approach could also be applied to the dysfunctional cholinergic system (Mesulam, 2004) in AD mice.

Another way to modulate specific populations of neurons is via chemogenetics (Box 1) (Armbruster et al., 2007). In addition to allowing for cell-type specificity and not requiring cranial implantations, which are needed in optogenetics, chemogenetics has the advantage of being able to trigger multiple sites simultaneously via systemic injection of a ligand. However, because the ligand might remain active for hours, temporal control of such stimulation is difficult. Chemogenetic stimulation combined with fMRI has not yet been used in AD research, but it has been used to examine fear-related brain networks in mice by selectively activating serotonin receptors in the amygdala (Gozzi et al., 2010), and to examine different regions connected to the ventral tegmental area of the rat brain (Roelofs et al., 2017). This suggests that the technique is feasible and worth applying to AD.

## Conclusions

The ability to reliably translate fMRI findings from the bench to the clinic has been hindered by a muddled AD etiology, the lack of a model that fully recapitulates human AD, and difficulties with performing fMRI in animals. Nevertheless, technological developments, such as optogenetics, chemogenetics and virtual reality setups, have the potential to push the boundaries of what can be achieved from performing fMRI studies in animal models of AD. In the coming years, they might also serve as catalysts for finally uncovering the origin of AD pathology in humans.

This Review raises important concerns and offers some potential solutions for fMRI study design. Future research will be necessary to address some of the outstanding problems. For example, most animal models of AD replicate FAD, although it is only representative of 5% of human cases. It is a priority, therefore, to generate reliable SAD models to advance AD research and treatment. Additionally, it is unclear what impact different neurotransmitter systems have on AD pathology, or which might be suitable therapeutic targets. Optogenetics and chemogenetics, when used to target specific neural populations, such as cholinergic neurons, will be useful in addressing this issue. Furthermore, the difficulty in performing task-based fMRI in rodents makes comparisons between AD animals and human patients more challenging. Implementation of behavioral tasks during rodent fMRI would allow for direct comparisons between new therapies tested in animal models and the associated symptoms observed in the clinic. Improvements in MRI-compatible behavior hardware and habituation protocols will aid in this endeavor. Lastly, future studies should focus not only on studying the pathology in AD animal models, but also on drawing direct comparisons to human AD research to see how well findings translate between the two. Our hope is that as the field advances and the experimental paradigms are refined, future studies will be able to finally uncover the key factors responsible for causing, treating and – one day – curing Alzheimer's disease.
